# Establishing the impact of WHO’s normative and standard-setting functions: a call for papers 

**DOI:** 10.2471/BLT.23.290829

**Published:** 2023-10-01

**Authors:** Lisa M Askie, Rebekah AL Thomas, Rok Ho Kim, Mubashar Sheikh, Jeremy Farrar

**Affiliations:** a Quality Assurance, Norms and Standards Department, World Health Organization, avenue Appia 20, 1211 Geneva 27, Switzerland.; b Science Division, World Health Organization, Geneva, Switzerland.

Normative leadership is a core function of the World Health Organization’s (WHO) mandate, as outlined in its founding principles.^1^ This leadership role is realized by developing evidence-based and ethically sound guidelines as well as other normative products that guide Member States in their public health decisions and actions, and by ensuring their recommendations are implemented.^2^ WHO exercises its capacity for normative leadership to influence the development of legal norms and health policy and practice within its Member States.

WHO develops a wide range of normative products including guidelines, standards, classifications, policy options, implementation guidance, learning materials, evaluation methods and research agendas. While the Organization has always had procedures and processes for developing its products, there have been criticisms that WHO guidance was not always based on systematically reviewed evidence, and that it did not use appropriate methodological expertise or adequately involve end-users, nor pay sufficient attention to implementation.^3^ In 2007, WHO responded by increasing the transparency and rigour of its guidelines with the introduction of the WHO Guidelines Review Committee and an accompanying guideline development handbook.^4^ Since 2017, WHO has committed to becoming data-driven, following internationally recognized methods and processes and fostering a new, outward-focused approach to partnerships. One key part of its transformation agenda, which sought to strengthen and expand the rigour of WHO’s normative leadership in science, innovation and research, was the establishment of the Science Division, led by WHO’s first-ever Chief Scientist, and including three new departments: Quality Assurance, Norms and Standards; Digital Health and Innovation; and Research For Health.^5^

In the past five years, WHO has initiated a major change process, driven by its current Thirteenth Global Programme of Work,^6^ the transformation agenda, and the need to respond to major global events including the coronavirus disease 2019 (COVID-19) pandemic and other crises. More recently, WHO has developed a comprehensive strategy to enhance its capacities and capabilities at country level, to ensure that its normative work drives measurable impact for all people more effectively.

Despite these initiatives, to date it is unclear whether these changes have improved WHO’s credibility and impact as a normative organization. Questions remain as to how successful WHO’s normative leadership role has been, how it can be further strengthened and how the impact of WHO’s work in countries should be measured and rated in the future. The recent COVID-19 pandemic exposed both the strengths of and the challenges to WHO’s normative leadership and global reach. While realizing the crucial role of WHO as a key directing and coordinating authority, the global health community has also witnessed the unprecedented rise of misinformation and mistrust in science. 

Considering the spotlight on WHO’s global normative leadership role during the pandemic,^7^ and looking to prepare for future threats such as the health effects of climate change and ongoing conflict situations, the *Bulletin of the World Health Organization* calls for papers to help shape and inform WHO’s mandate going forward. Topics of interest include: where has WHO succeeded in its normative leadership role? In what specific areas has it successfully shaped global health, and why were these initiatives successful? Where has WHO normative guidance been less impactful? If so, why was this the case, and what lessons can be learnt to improve impact in the future? What does the future of WHO’s normative function look like, particularly in the context of digital technology and artificial intelligence? What aspects of the Organization’s mandate, structure, function and administration need to be further strengthened or changed?

The *Bulletin* welcomes contributions from all stakeholders including public health decision-makers, researchers, and civil society and community representatives. Articles that propose innovative but feasible ways by which WHO can further strengthen its normative leadership and guidance role, are encouraged.

The deadline for submissions is 1 March 2024. Manuscripts should be submitted in accordance with the *Bulletin*’s guidelines for contributors (available at: https://www.who.int/publications/journals/bulletin/ contributors/guidelines-for-contributors) and the cover letter should mention this call for papers. This theme issue will be published in the October 2024 issue of the *Bulletin*, to coincide with the annual World Evidence-Based Health Care Day on 20 October 2024.
